# Remote Home Monitoring of Continuous Vital Sign Measurements by Wearables in Patients Discharged After Colorectal Surgery: Observational Feasibility Study

**DOI:** 10.2196/45113

**Published:** 2023-05-05

**Authors:** Jobbe P L Leenen, Vera Ardesch, Gijsbert Patijn

**Affiliations:** 1 Connected Care Center Isala Zwolle Netherlands; 2 Department of Surgery Isala Zwolle Netherlands; 3 Isala Academy Isala Zwolle Netherlands; 4 Flexpool General Wards Department of Care Support Isala Zwolle Netherlands

**Keywords:** telemedicine, remote monitoring, home monitoring, virtual care intervention, colorectal surgery, continuous vital signs monitoring, wearable wireless monitoring, clinical deterioration, readmission, virtual care, online intervention, e-health, remote monitoring, telehealth, vital signs

## Abstract

**Background:**

Hospital stays after colorectal surgery are increasingly being reduced by enhanced recovery and early discharge protocols. As a result, postoperative complications may frequently manifest after discharge in the home setting, potentially leading to emergency room presentations and readmissions. Virtual care interventions after hospital discharge may capture clinical deterioration at an early stage and hold promise for the prevention of readmissions and overall better outcomes. Recent technological advances have enabled continuous vital sign monitoring by wearable wireless sensor devices. However, the potential of these devices for virtual care interventions for patients discharged after colorectal surgery is currently unknown.

**Objective:**

We aimed to determine the feasibility of a virtual care intervention consisting of continuous vital sign monitoring with wearable wireless sensors and teleconsultations for patients discharged after colorectal surgery.

**Methods:**

In a single-center observational cohort study, patients were monitored at home for 5 consecutive days after discharge. Daily vital sign trend assessments and telephone consultations were performed by a remote patient-monitoring department. Intervention performance was evaluated by analyzing vital sign trend assessments and telephone consultation reports. Outcomes were categorized as “no concern,” “slight concern,” or “serious concern.” Serious concern prompted contact with the surgeon on call. In addition, the quality of the vital sign data was determined, and the patient experience was evaluated.

**Results:**

Among 21 patients who participated in this study, 104 of 105 (99%) measurements of vital sign trends were successful. Of these 104 vital sign trend assessments, 68% (n=71) did not raise any concern, 16% (n=17) were unable to be assessed because of data loss, and none led to contacting the surgeon. Of 62 of 63 (98%) successfully performed telephone consultations, 53 (86%) did not raise any concerns and only 1 resulted in contacting the surgeon. A 68% agreement was found between vital sign trend assessments and telephone consultations. Overall completeness of the 2347 hours of vital sign trend data was 46.3% (range 5%-100%). Patient satisfaction score was 8 (IQR 7-9) of 10.

**Conclusions:**

A home monitoring intervention of patients discharged after colorectal surgery was found to be feasible, given its high performance and high patient acceptability. However, the intervention design needs further optimization before the true value of remote monitoring for early discharge protocols, prevention of readmissions, and overall patient outcomes can be adequately determined.

## Introduction

Colorectal surgery is known for high complication and readmission rates [1-5]. In the last decade, enhanced recovery programs, such as the Enhanced Recovery After Surgery (ERAS) program, have been adopted widely and have resulted in significantly shorter hospital lengths of stay, with discharge as early as postoperative day 1 or 2 [6-8]. Serious postoperative complications such as anastomotic leak, abscess, ileus, thrombosis, or surgical site infection may therefore manifest themselves in the home setting [5,9]. Follow-up of these patients is generally limited to outpatient clinic visits that do not take place until several weeks after discharge. Late recognition of signs and symptoms by patients may cause delayed detection and lead to inferior clinical outcomes and readmissions [1,5,10].

Virtual care interventions such as remote patient monitoring with mobile health apps have the potential to further reduce lengths of hospital stay and prevent unnecessary readmissions, reduce emergency department visits, and help mitigate increasing nursing staff shortages and overall health care costs [11-13]. Such interventions require only limited investment in time and money if deployed at sufficient scale [14]. Virtual care interventions may therefore help to remove barriers to further expand early discharge protocols.

Recent technological advances have enabled continuous vital sign monitoring by wearable wireless sensor devices [15,16]. These devices have been shown to be able to accurately detect deviating vital sign trends [15,17]. Such technology may allow patients to safely recover at home while being carefully monitored with the intention to capture possible clinical deterioration at an early stage.

Several studies have demonstrated the feasibility of continuous vital sign monitoring in the hospital with wireless wearables [18-21], but there are only a few studies on remote home monitoring after discharge, and these have mostly been limited to intermittent monitoring of vital signs [11,22]. Only one study showed that continuous vital sign monitoring was technically feasible and well accepted by patients discharged after esophageal surgery [23]. It is unknown if a comparable intervention is feasible in patients after colorectal surgery. Therefore, the objective of this study was to determine the feasibility of a virtual care intervention consisting of continuous vital sign monitoring with wearable wireless sensors and teleconsultations supported by a central, remote patient-monitoring department for patients discharged after colorectal surgery.

## Methods

### Study Design and Setting

A single-center observational cohort study was conducted in May and June 2022 in a 1250-bed teaching hospital in the Netherlands. This study is reported in accord with the STROBE (Strengthening the Reporting of Observational Studies in Epidemiology) guidelines [24].

### Participants

Patients scheduled for elective colorectal resection in May and June 2022 were approached for consent for the remote monitoring intervention during the preadmission call by a nurse. Inclusion criteria were as follows: age ≥18 years, elective colorectal resection, primary anastomosis, admission to the participating surgical ward, an uncomplicated clinical course (duration of admission <7 days), presence of an adequate caregiver at home, possession of a mobile phone, and no cognitive impairments. Exclusion criteria were as follows: unable to wear a continuous monitoring device due to a pacemaker or allergy, no desire for treatment (or no desire for referral) in the event of clinical deterioration, cognitive impairment at discharge, discharge to a rehabilitation or nursing home, physical limitations that would hinder participation, and insufficient command of the Dutch language.

### Remote Home Monitoring Intervention

**Figure 1:**
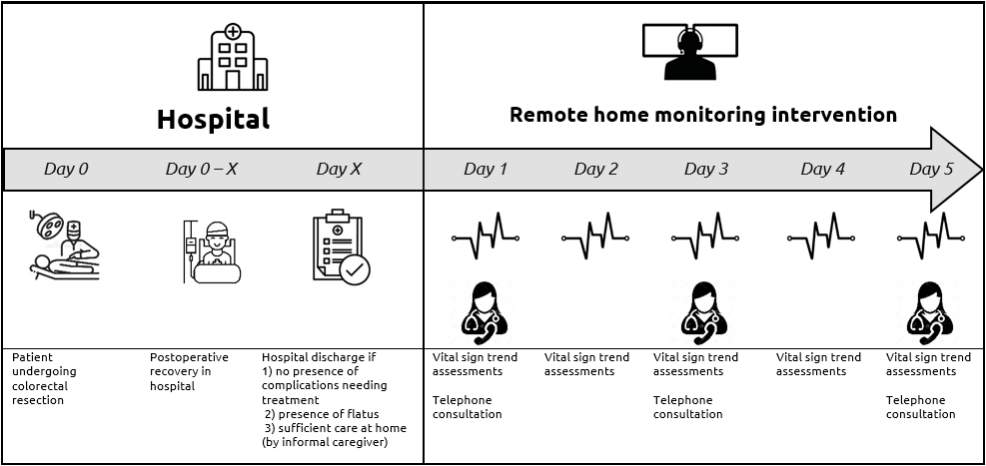
Overview of the care path for included patients.

The remote home monitoring intervention was developed based on a previous study [25] that evaluated in-hospital continuous vital sign monitoring developed in cooperation with an abdominal surgeon, ward nurses, and nurses at the remote monitoring department of the hospital. The intervention consisted of 5 days of follow-up with 3 consultations by phone (every other day) and daily evaluation of vital sign trends with the wearable sensor (Figure 1).

The consultations by phone were performed by the specialized cardiac care nurses at the remote monitoring department, who were dedicated to and had expertise in virtual care and remote monitoring of patients. The department is operated every day of the week from 8 AM to 11 PM. The consultation consisted of an assessment of the patient’s condition regarding pain symptoms, wound condition, stool, and nausea. In addition, vital sign trends measured by the wearable sensor were assessed daily and at the time of the telephone consultation.

Vital signs of patients were measured by the Conformité Européene–marked Healthdot sensor and Intellivue Guardian Solution (IGS) software system (Philips). The wireless sensor is a previously validated patch worn on the patient’s chest that continuously records heart rate (HR) in beats per minute (bpm) and respiratory rate (RR) in respirations per minute (rpm) with a battery life of 14 days (Multimedia Appendix 1). The 2 vital sign measurements are transmitted wirelessly every 5 minutes through a long range, low power Internet of Things (LoRa) connection to the IGS software. Within the IGS software, vital sign trends are visualized and, complementary to the hospital Modified Early Warning Score (MEWS) protocol, the Deel EWS (D-EWS; *Deel* is Dutch for “partial”), a partial MEWS score, was aggregated every hour to promote adequate interpretation (Multimedia Appendix 2). The scores are based on the thresholds for HR and RR when sufficient data were present. There were no active alarms generated to the nurses when scores deviated. If any acute help was needed, patients were informed to call emergency services.

### Study Procedures

Before the start of the study, nurses at the remote patient monitoring department were educated by the project manager (JPLL) and a colorectal surgeon (GP) in a 1.5-hour information session about colorectal resections, the most common complications, and nursing care for these patients. Moreover, the trend assessment used in this study was informed by case studies. In a 2-month period (May-June 2022), patients scheduled for colorectal resection were approached by the ward nurse, received information about the home monitoring intervention, and were asked to provide verbal informed consent during a preoperative telephone consultation. Directly postoperatively, the sensor was attached to the patient at the ward and in-hospital continuous monitoring was performed. When the in-hospital postoperative course was uncomplicated, the patient was eligible for home monitoring in this study. At discharge, the ward nurse asked the remote monitoring department to start the monitoring.

### Data Collection

Baseline characteristics were collected, including age, sex, height, weight, BMI, American Society of Anesthesiologists (ASA) score, Charlson Comorbidity Index [26], type of colorectal surgery and procedure (open or laparoscopic), indication for surgery (malignant or benign), length of hospital stay in days, MEWS at discharge, heart rate and respiratory rate at discharge, and readmission ≤30 days. The primary end point was feasibility, defined by the following 3 outcomes: intervention performance, quality of vital sign data, and patient experience.

### Intervention Performance

For each patient, we assessed whether the intervention was carried out according to protocol with daily vital sign assessment and telephone consultations. Daily vital sign trend assessments were scored as 0 (no cause for concern), 1 (slight concern), and 2 (serious concern). A score of 1 resulted in a wait-and-see approach and a score of 2 resulted in contact with the surgeon on call. An X was scored if the nurse was unable to assess the vital sign trend because of insufficient vital sign data. After the telephone consultation, the same scoring was done by assessing pain (rated by the Numeric Rating Scale), wound infection (redness/pain of the wound area), and experience of nausea (yes or no) and defecation (yes or no). Additionally, discrepancies in scoring between vital sign trend assessments and telephone consultations were registered. Finally, the time spent for the intervention and for each consultation was registered in time frames of 5 minutes.

### Quality of the Vital Sign Data

The quality of vital sign data was defined as the proportion of trend assessments that were impossible to perform, as well as completeness of the vital sign data, the generated D-EWS scores, heart rate and respiration measurements, and the distribution of data gaps based on their duration: <1 hour, 1 to 4 hours, 4 to 8 hours, 8 to 16 hours, and 16 to 24 hours.

### Patient Experience

Patient experience was measured at the end of the remote monitoring intervention by an 11-item questionnaire. Two questions assessed the overall experience of the patient and informal caregiver (when present) with a 10-point Likert scale, 7 questions assessed specific domains with a 5-point Likert scale, and 2 questions had open answers (Multimedia Appendix 3).

### Statistical Analysis

Descriptive statistics were used to evaluate patient demographics and to assess the feasibility of home monitoring. Normally distributed continuous data are presented as the mean (SD). Likewise, nonnormally distributed data are presented as the median (IQR). Normality was determined with the Kolmogorov-Smirnov test and visually by a quantile-quantile plot and histogram. Nominal data were presented as frequencies (n) and percentages (%). All data were analyzed with SPSS Statistics (version 26; IBM Corp). Formal sample size calculation was challenging given the observational feasibility study design, but a sample in the range of 20 to 25 is considered adequate for this type of study [27]. Therefore, we aimed to include 20 patients and obtain data from 100 home monitoring days.

### Ethical Considerations

The Medical Ethics Review Committee of Isala waived the need for ethical approval (210414). The study was conducted in accordance with the Declaration of Helsinki. Written informed consent was obtained from each participant in the study.

## Results

### Study Characteristics

A total of 25 patients were screened, of whom 23 (92%) agreed to participate. After 1 patient dropped out and 1 patient had in-hospital complications, a total of 21 patients participated in the study. The mean age was 67 (SD 13) years, and 8 (38%) participants were male. The mean length of stay was 4.4 (SD 2.2) days, and hemicolectomies were mostly right sided (n=8, 38%). Three patients (14%) were readmitted to the hospital within 30 days, but there were no alterations in clinical decision-making or the clinical course based on the intervention (Multimedia Appendix 4). A full description of the participants is given in Table 1.

**Table 1:** Patient characteristics (n=21).

Characteristic		Value
Age (years), mean (SD)		67 (13)
Sex (male), n (%)		8 (38)
Height (cm), mean (SD)		173 (9)
Weight (kg), mean (SD)		78 (15)
BMI (kg/m^2^), mean (SD)		25.9 (4.2)
**American Society of Anesthesiologists class, n (%)**		
	1-2	15 (71)
	3-4	6 (29)
Charlson Comorbidity Index, mean (SD)		3.9 (2.3)
**Type of surgery, n (%)**		
	Right hemicolectomy	8 (38)
	Sigmoid resection	6 (29)
	Abdominal perianal resection	3 (14)
	Wig resection colon	2 (10)
	Ileocecal resection	1 (5)
	Low anterior resection	1 (5)
Laparoscopic procedure, n (%)		20 (95)
**Indication for surgery, n (%)**		
	Malignant	16 (76)
	Benign	5 (24)
Length of hospital stay (days), mean (SD)		4.4 (2.2)
**Modified Early Warning Score at discharge, n (%)**		
	0	8 (38)
	1	12 (57)
	2	1 (5)
Heart rate at discharge (bpm), mean (SD)		81 (13)
Respiratory rate at discharge (rpm), mean (SD)		14 (1)
Readmission <30 days, n (%)		3 (14)

### Intervention Performance

Of 21 patients, 1 received only telephone consultations, because the vital sign patch was removed by accident on the ward by a ward nurse. The median time spent for the home monitoring was 40 (IQR 35-40) minutes per patient, and 49% (51/104) of the daily home monitoring checks took 5 minutes or less for the nurses to perform (Table 2). A total of 104 (99%) vital sign trend assessments were performed. The majority (71/104, 68%) of assessments did not raise concerns and 16% (17/104) could not be assessed because of excessive data loss. Nonetheless, no cases were found where the nurse was seriously concerned about a vital sign trend.

Considering the telephone consultations, 62 of 63 (98%) were performed because in one case, the patient could not be reached. The median pain score was 3 (IQR 2-4) at the first consultation and 2 (IQR 1-2) at the third consultation. In 1 of 62 consultations (1.7%) the wound assessment was painful and resulted in a score of 1 (slightly concerned) for which a wait-and-see approach was taken. In 6 of 62 consultations (10%), the patients had not yet defecated. In the majority of consultations (53/62, 85%), no concerns were raised. Only 1 of 62 consultations (1.7%) resulted in contact with the surgeon on call, due to blood loss during defecation, for which a wait-and-see policy was agreed to be followed.

**Figure 2:**
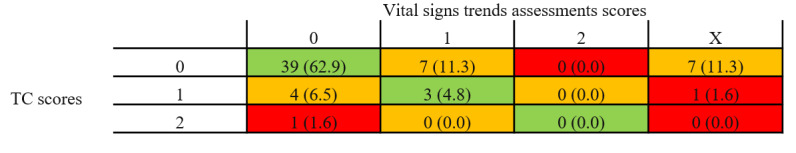
Agreement between trend assessments and telephone consultations (n=62). Values are shown as n (%). Green indicates agreement; orange indicates that there was no agreement and a wait-and-see approach was taken; red indicates that there was no agreement. X indicates that trend data were lacking. TC: telephone consultation.

Considering agreement, 42 (68%) of 62 telephone consultations were in agreement with the trend assessments, and in 8 (13%) telephone consultations, agreement was not possible because of a lack of trend data. Only once, the telephone consultation raised concern but the trend assessment did not (Figure 2).

**Table 2:** Intervention performance.

Variable		Value
Total time spent by nurses on assessments per patient (minutes), median (IQR)		40 (35.0-40.0)
**Time taken by nurses to complete** **daily home monitoring checks (n=104), n (%)**		
	≤5 minutes	51 (49)
	>5 to ≤10 minutes	47 (45)
	>10 to ≤15 minutes	5 (5)
	25 minutes	1 (1)
**Result of vital sign assessment (n=104), n (%)**		
	No concern	71 (68)
	Slight concern	16 (15)
	Concern	0 (0)
	Unable to assess	17 (16)
**Result of telephone consultations (n=62), n (%)**		
	No concern	53 (85)
	Slight concern	8 (13)
	Concern	1 (2)
**Numeric Rating Scale pain** **score (range 0-10)** **, median (IQR)**		
	Day 1	3 (2-4)
	Day 3	2 (1-4)
	Day 5	2 (1-2)
Wound assessments of rubor/dolor (n=21), n (%)		1 (5)
Absence of defecation (n=63), n (%)		6 (10)

### Quality of the Vital Sign Data

Monitoring data were available for 20 patients for a total of 2347 hours of vital sign data with a median of 116.6 (IQR 115.5-118.7) hours per patient (Table 3). Data gaps of 30 minutes or less occurred in 95.2% (12,402/13,039) of measurements. Gaps between 30 and 60 minutes were detected in 2.6% (338/13,039) of measurements, and gaps between 1 and 16 hours were detected in 2.3% (299/13,039) of measurements. This resulted in an overall completeness of vital sign trends of 46.3% (range 5%-100%).

Of the 13,039 completed measurements, HR measurements contained a median of 15.6% (IQR 9.7%-35%) artifact data and RR measurements contained a median of 21.8% (IQR 9.3%-25.9%) artifact data. A total of 215 D-EWS scores were calculated based on the data, of which 40 (18.6%) were 3 or higher.

**Table 3:** Quality of vital sign data (total vital sign measurements=13,039).

Variable		Value
Monitoring time (hours), median (IQR)		116.6 (115.5-118.7)
Monitoring time (minutes), median (IQR)		6995 (6929-7124)
Heart rate (beats per minute), mean (SD)		74 (12)
Respiratory rate (breaths per minute), mean (SD)		17 (2)
**D-EWS^a^(n=1153), n (%)**		
	0	215 (18.6)
	1	701 (60.8)
	2	197 (17.1)
	3 or higher	40 (3.5)
Vital sign measurements per patient, median (IQR)		519 (284-1085)
Completeness of vital sign data, median (IQR)		36.6 (20.1-77.7)
Artifacts in heart rate measurements, median (IQR)		15.6 (9.7-35.0)
Artifacts in respiratory rate measurements, median (IQR)		21.8 (9.3-25.9)
**Data gaps (n=13,039), n (%)**		
	0-30 minutes	12,402 (95.1)
	30-60 minutes	338 (2.6)
	60-120 minutes	169 (1.3)
	2-4 hours	91 (0.7)
	4-8 hours	26 (0.2)
	8-16 hours	13 (0.1)

^a^D-EWS: Deel Early Warning Score (*deel* is Dutch for “partial”).

### Patient Experience

Twenty patients returned the questionnaire (Table 4). The median satisfaction scores were 8 (IQR 7-9) of 10 for patients and 8 (IQR 5-8) of 10 for caregivers (n=7). The majority of patients (n=18, 90%) found the sensor comfortable to wear, although several remarks were made about experiences of discomfort during lateral sleep because of the rigidity of the sensor’s material (Multimedia Appendix 5). Further, the majority of patients liked to have insight in vital sign trends (n=18, 90%) and to have telephone consultations (n=17, 85%); a majority also found contact by phone adequate (n=17, 85%). Two patients (10%) felt safer with home monitoring, whereas 7 (35%) were neutral, and 11 (55%) did not feel safer. For future home monitoring interventions, 70% of patients agreed that they would want to be discharged from the hospital at an earlier stage if there were a home monitoring intervention. Possible reasons for this, based on the remarks section of the questionnaire, were that patients experienced better recovery in the home setting overall and because they experienced sleep interruptions at night while being admitted in the hospital (Multimedia Appendix 5).

**Table 4:** Results of the patient survey.

Question		Median score (IQR)	Responses, n (%)		
			Disagree^a^	Neutral^b^	Agree^c^
**Likert-scale items (range 1 to 10)**					
	In general, how did you experience the home monitoring period? (n=20)	8 (7-9)	0 (0)	2 (10)	18 (90)
	How did your informal caregiver experience the home monitoring period overall? (n=7)	8 (5-8)	0 (0)	2 (29)	5 (71)
**Likert-scale items (range 1 to 5; n=20)**					
	I found the wearable sensor comfortable.	5 (4-5)	2 (10)	1 (5)	17 (85)
	I liked that healthcare professionals could see my vital signs (heart rate, breathing) on a daily basis.	5 (4.3-5)	1 (5)	1 (5)	18 (90)
	I need to have insight into my vital signs measurements (heartbeat, breathing).	4 (3-5)	0 (0)	8 (40)	12 (60)
	I liked the telephone contact with the healthcare professionals.	5 (4-5)	0 (0)	3 (15)	17 (85)
	The telephone contacts were sufficient.	4 (4-5)	2 (10)	1 (5)	17 (85)
	I felt safer with the home monitoring than if I had not had it.	4 (3.3-5)	11 (55)	7 (35)	2 (10)
	If in the future you were allowed to go home a day earlier with home monitoring, would you want to?	4 (3-5)	0 (0)	6 (30)	14 (70)

^a^Represents a score of 1 to 4 on the 10-item Likert scale or 1 to 2 on the 5-item Likert scale.

^b^Represents a score of 5 on the 10-item Likert scale or 3 on the 5-item Likert scale.

^c^Represents a score of 6 to 10 on the 10-item Likert scale or 4 to 5 on the 5-item Likert scale.

## Discussion

### Principal Findings

In this study, we found that a remote home monitoring intervention for colorectal surgical patients consisting of vital sign trend assessments and telephone consultations performed by nurses at a remote home monitoring department was feasible. The intervention performance and patient acceptability were high, whereas the quality of vital sign data was still variable.

### Intervention Performance

Our results show that 5 days of follow-up with vital sign trend assessments combined with telephone consultations could be successfully performed. The agreement between these 2 methods of patient assessment was adequate (nearly 70%). In this small cohort, the majority of assessments did not raise concerns about patient recovery, and no subsequent interventions were required, but this needs confirmation in a larger cohort. Given the limited time spent by nurses delivering the intervention, this type of postdischarge monitoring may become cost-efficient when operated at sufficient scale [14], especially if it proves to facilitate even shorter hospital stays and prevent readmissions.

To achieve this, further design optimizations are first needed. Telephone consultations may be replaced by a symptom questionnaire and self-registration in a mobile digital app by patients themselves, which promotes self-management and reduces workload for the monitoring nurse [28].

Importantly, the optimal measurement frequency for the detection of deviations in a timely manner among postdischarge surgical patients at home is still unknown [17,29]. A limited number of measurements per day rather than continuous measurements may be sufficient for the timely detection of deterioration for this patient category. Also, daily assessment of vital sign trends with the D-EWS score may not be needed for the majority of patients, as these types of early warning scores are designed to provide alerts for serious clinical deterioration, which is rare postdischarge in the home setting. Comparative studies of continuous versus intermittent vital sign measurements are needed to further investigate the optimal intervention for this patient category.

### Quality of Vital Sign Data

Besides the design of the intervention, the technology of wearable wireless devices for continuous vital sign home monitoring also needs further optimization. In our study, trends were generated by performing vital sign measurement every 5 minutes. Despite the relatively low amount of measurement artifacts and data gaps, completeness of data was still low (median 37% per patient). In particular, the highly variable completeness was partly caused by data gaps longer than 4 hours, which hampered adequate trend assessment by the nurses. A possible explanation for this data loss is that the connectivity of the wireless LoRa network is currently influenced by external factors, such as indoor coverage, which can be improved by installing an amplifier for home monitoring.

### Patient Experiences

Patient acceptance was very high in this study, showing that patients were content to participate in this type of care after discharge. This may be explained by the limited time investment required of patients for the intervention and the additional patient-nurse interaction provided by telephone consultations [30]. Furthermore, the majority of patients also indicated that they would not mind being discharged earlier if they received this remote home monitoring intervention as standard care, which shows this type of home monitoring intervention could be incorporated into early discharge protocols. In addition, when these vital sign measurements are recorded and submitted by the patients themselves instead of by automated technology, patients may participate more actively in recovery management, which is associated with better outcomes [31-33] and may encourage a more sustainable, healthier life style [11,31]. On the other hand, contextual factors, such as circadian rhythm and patient mobilization, are not taken into account with single measurements made by patients themselves. Use of a wearable sensor for continuous monitoring may provide a more holistic impression of activity and patients’ circadian rhythm, but the significant associated cost of the sensors must be considered [14].

### Comparison With Other Work

Previous literature on this topic is scarce, but our results on intervention performance and compliance are in line with 2 previous home monitoring interventions that were comparable to ours [23,34]. Our findings are in contrast with a previous systematic review showing that remote monitoring interventions using mobile health were associated with improved surveillance, earlier detection of complications, and more timely interventions, preventing further clinical decline [11]. However, the designs of the postoperative home monitoring interventions that were reviewed were highly variable, and the interventions did not use continuous vital sign measurements with wearable devices. With regard to patient acceptability and satisfaction, previously reported comparable interventions found similarly high rates [23,34,35].

### Strengths and Limitations

This is one of the first studies to examine the feasibility of a home monitoring intervention for colorectal surgery patients using an available certified wearable sensor, software, and infrastructure. However, when interpreting the findings of this study, some limitations should be considered. First, although our primary aim was to determine feasibility, the generalizability of our results is limited because of the study design and relatively small sample size. Research on adequate and timely detection of clinical deterioration with remote home monitoring interventions (potentially preventing readmissions) requires large cohorts to assess the true benefits. Second, assessment of continuous vital sign trend data specifically for perioperative patients was a novel method for the monitoring care professionals, which may have influenced the adequacy of the assessments, even though the care professionals were specialized and trained in remote patient monitoring. Third, we used a self-developed questionnaire to assess patient experiences, since a validated questionnaire suitable for this type of intervention was not available. Finally, we did not include cost as an end point in this feasibility study. An early assessment of costs may be relevant for future optimization of such interventions, especially as wearable sensor technology is still associated with significant up-front investments and device cost.

### Future Directions

Although these are promising initial results, further studies on the design of a remote home monitoring intervention for this patient category are needed before the true value for early discharge protocols, prevention of readmissions, and patient outcomes can be adequately determined. First, future research is needed to explore the needs of patients and health care professionals for each care pathway regarding telephone consultations and vital sign measurements. Consideration should be given to replacing telephone consultations with indirect digital contact through a mobile app for monitoring and coaching to achieve even more efficient care. Subsequently, the expansion of vital sign measurements to include other parameters, such as body temperature and mobility, may be relevant to capture the full status of the patient. In addition, the true added value of continuous versus intermittent vital sign measurements and the optimal frequency and timing of teleconsultations need to be determined for each patient category. Notifications generated by a combination of vital sign measurements and symptoms could assist monitoring professionals in clinical decision-making. Ultimately, optimal home monitoring protocols may eventually be personalized depending on surgery type, postoperative course, and individual patient characteristics, whereby deviating vital sign trend detection will be automated by algorithms that will support the monitoring department staff.

### Conclusion

We found that a remote home monitoring intervention for colorectal surgical patients with wearable wireless sensors and telephone consultations was feasible, considering the high intervention performance and high patient acceptability. However, the design of a remote home monitoring intervention for this patient category should be further optimized before its true value for early discharge protocols, prevention of readmissions, and patient outcomes can be adequately determined.

## Appendix

Multimedia Appendix 1 The Philips Healthdot wearable sensor.

Multimedia Appendix 2 Thresholds of the partial Early Warning (D-EWS) scores.

Multimedia Appendix 3 Patient questionnaire.

Multimedia Appendix 4 Case description of readmissions.

Multimedia Appendix 5 Remarks of patients at the questionnaire (translated from Dutch).

## Abbreviations

ASA: American Society of Anesthesiologists
D-EWS: Deel Early Warning Score
ERAS: Enhanced Recovery After Surgery
HR: heart rate
IGS: Intellivue Guardian Solution
LoRa: long range, low power Internet of Things
RR: respiratory rate

## Data Availability

The data sets generated during and/or analyzed during this study are available from the corresponding author on reasonable request.
