# Supplementary material for: Remote Home Monitoring of Continuous Vital Sign Measurements by Wearables in Patients Discharged After Colorectal Surgery: Observational Feasibility Study
Source: JMIR Perioper Med. 2023 May 5;6:e45113. doi: 10.2196/45113 (PMC10199380; doi:10.2196/45113)
Supplement: Multimedia Appendix 1 [file periop_v6i1e45113_app1.docx]

**MULTIMEDIA APPENDIX 1: The Philips Healthdot wearable sensor**


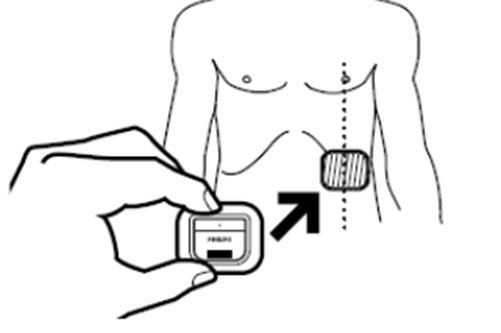


Reprinted from Philips Electronic Nederland BV under a CC BY license, with permission from Philips Electronic Nederland BV, original copyright 2020

This is a Multimedia Appendix to a full manuscript published in the JMIR Perioperative Medicine. For full copyright and citation information see http://dx.doi.org/10.2196/jmir. 45113
