# Supplementary material for: Remote Home Monitoring of Continuous Vital Sign Measurements by Wearables in Patients Discharged After Colorectal Surgery: Observational Feasibility Study
Source: JMIR Perioper Med. 2023 May 5;6:e45113. doi: 10.2196/45113 (PMC10199380; doi:10.2196/45113)
Supplement: Multimedia Appendix 2 [file periop_v6i1e45113_app2.docx]

**MULTIMEDIA APPENDIX 2: Thresholds of the partial Early Warning (D-EWS) scores**

|  | **D-EWS score** | | | | | |
| --- | --- | --- | --- | --- | --- | --- |
| Score | 2 | 1 | 0 | 1 | 2 | 3 |
| Heart rate (HR) | <40 | 40-50 | 51-100 | 101-110 | 111-130 | >130 |
| Respiratory rate (RR) | <9 |  | 9-14 | 15-20 | 21-30 | >30 |

This is a Multimedia Appendix to a full manuscript published in the JMIR Perioperative Medicine. For full copyright and citation information see http://dx.doi.org/10.2196/jmir. 45113
