# Supplementary material for: Remote Home Monitoring of Continuous Vital Sign Measurements by Wearables in Patients Discharged After Colorectal Surgery: Observational Feasibility Study
Source: JMIR Perioper Med. 2023 May 5;6:e45113. doi: 10.2196/45113 (PMC10199380; doi:10.2196/45113)
Supplement: Multimedia Appendix 3 [file periop_v6i1e45113_app3.docx]

**MULTIMEDIA APPENDIX 3: Patient questionnaire**

1. In general, how did you experience the home monitoring period? (scale 1-10)
2. How did your informal caregiver experience the home monitoring period overall? (scale 1-10)
3. I found the wearable sensor comfortable. (scale 1-5)
4. I liked that healthcare professionals could see my vital signs (heart rate, breathing) on a daily basis. (scale 1-5)
5. I need to have insight into my vital signs measurements (heartbeat, breathing). (scale 1-5)
6. I liked the telephone contact with the healthcare professionals. (scale 1-5)
7. The telephone contacts were sufficient. (scale 1-5)
8. I felt safer with the home monitoring than if I had not had it. (scale 1-5)
9. If in the future you were allowed to go home a day earlier with home monitoring, would you want to? (scale 1-5)
10. Please explain (open-end question)
11. Free space for remarks (open-end question)

This is a Multimedia Appendix to a full manuscript published in the JMIR Perioperative Medicine. For full copyright and citation information see http://dx.doi.org/10.2196/jmir. 45113
