# Supplementary material for: Remote Home Monitoring of Continuous Vital Sign Measurements by Wearables in Patients Discharged After Colorectal Surgery: Observational Feasibility Study
Source: JMIR Perioper Med. 2023 May 5;6:e45113. doi: 10.2196/45113 (PMC10199380; doi:10.2196/45113)
Supplement: Multimedia Appendix 4 [file periop_v6i1e45113_app4.docx]

**Multimedia Appendix 4: Case description of readmissions**

| **Table 1: Assessments studies of individual readmitted patients** | | | | | | | | | |
| --- | --- | --- | --- | --- | --- | --- | --- | --- | --- |
| Day of home monitoring | 1 | | 2 | 3 | | 4 | 5 | | |
|  | VS | TC | VS | VS | TC | VS | | VS | TC |
| Patient 1 | 0 | 0 | 0 | X | 1 | 0 | | X | 0 |
| Patient 2 | 1 | 1 | 1 ^a^ | 0 | 0 | 0 | | 0 | 0 |
| Patient 3 | 0 | 0 | 0 | 0 | 0 | 0 | | Readmitted | |
| Abbreviations: VS: vital signs trends assessments, TC: telephone consultation, ^a^ readmitted for <24 hours | | | | | | | | | |

**Patient #1**

The first patient (female, 53 years old, ileocecal resection), was readmitted for 9 days 23 days after end of the home monitoring because of an abscess treated with antibiotics (Clavien Dindo Complication Classification II). The vital signs trends showed deviation in heartrate in the morning (on day 3 and 5) most likely related to exercise during personal care in the morning for which score 1 was assigned (Table 1).


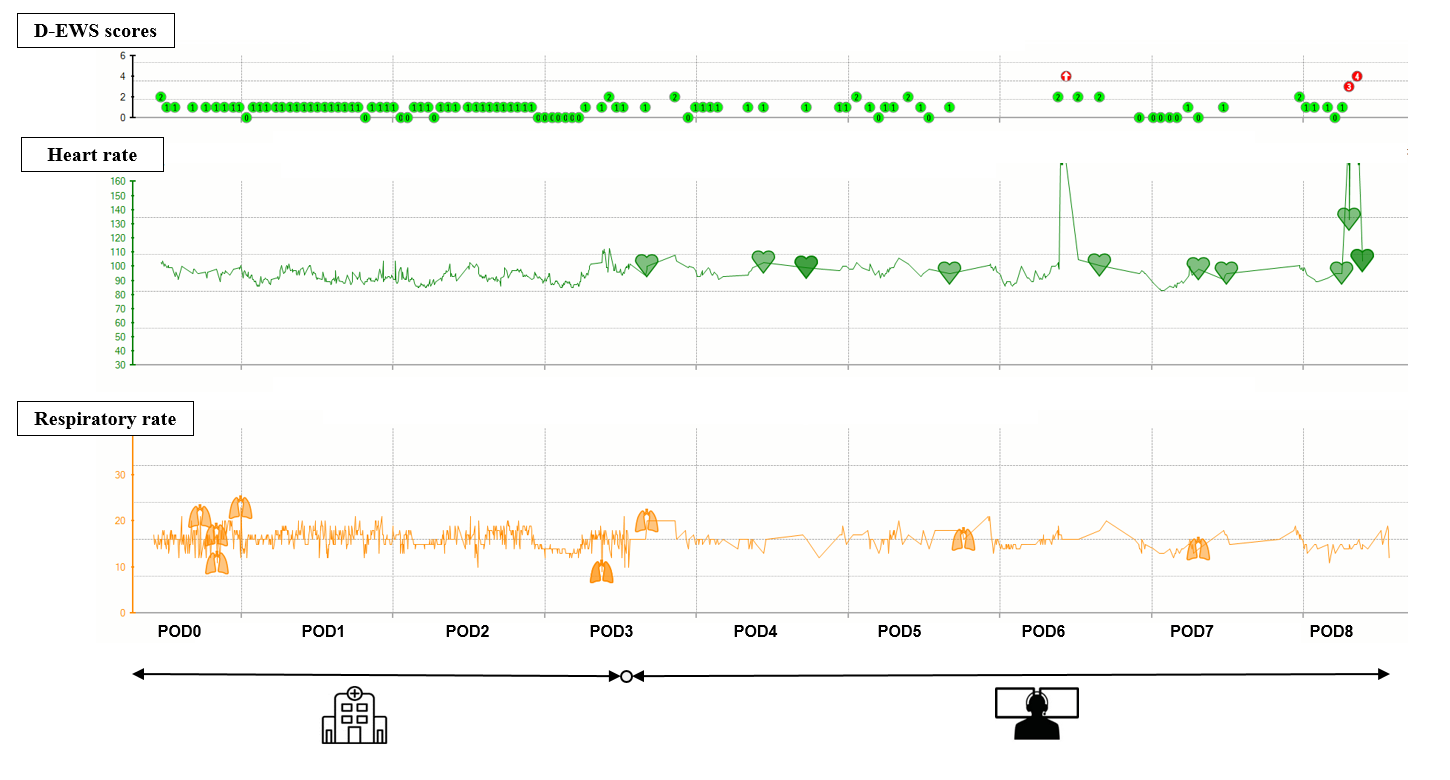


**Patient #2**

The second patient (female, 60 years old, sigmoid resection), was readmitted for only 1 day on the third day of home monitoring because of clinical observation with a sub febrile body temperature and free air on an X-ray (Table 1) (Clavien Dindo Complication Classification I).The vital signs trends showed deviation in heartrate and respiratory rate resulting in D-EWS scores of 4 and 3 a day before readmission. However, the trend assessment resulted in a score of 1 so the home monitoring did not affect the clinical decision making related to the readmission.

**
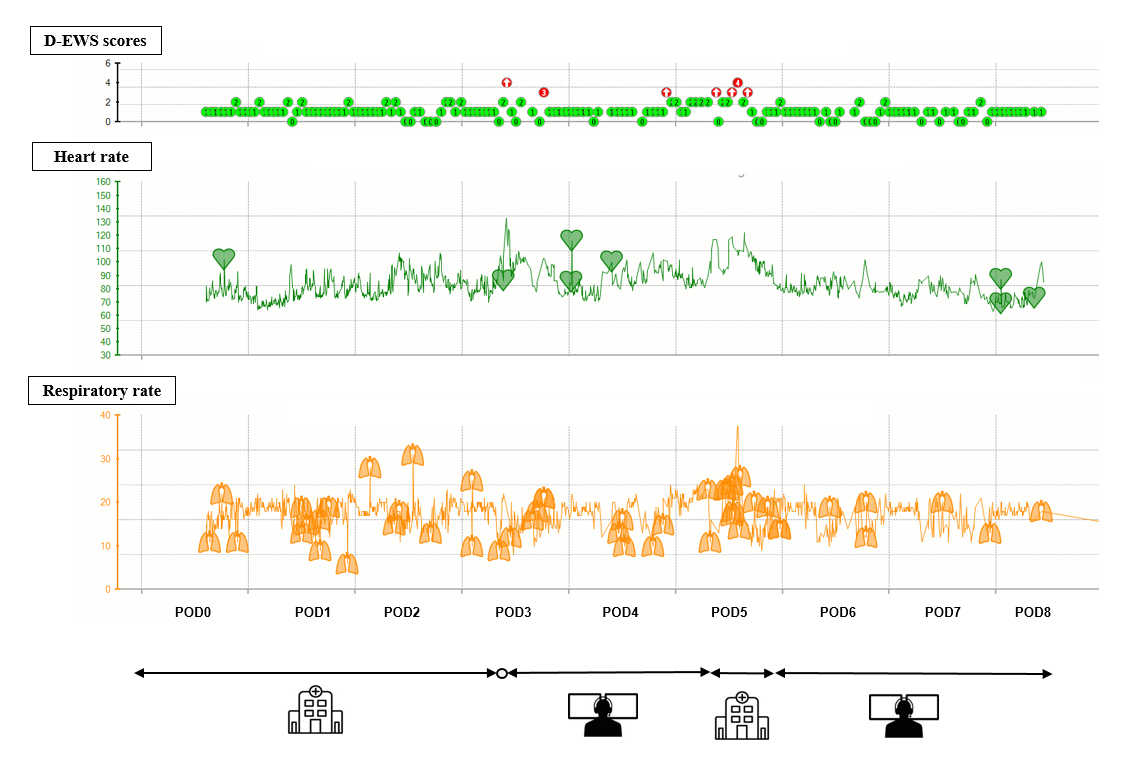
**

**Patient #2**

The third patient (male, 60 years old, abdominal perianal resection) was readmitted for 3 days on the fifth day of home monitoring because of the clinical observation of rectal blood loss (Clavien Dindo Complication Classification I) (Table 1). The vital sign trends did not deviate, and also the telephone consultation in a scores of 0 so home monitoring did not play any role in the readmission.


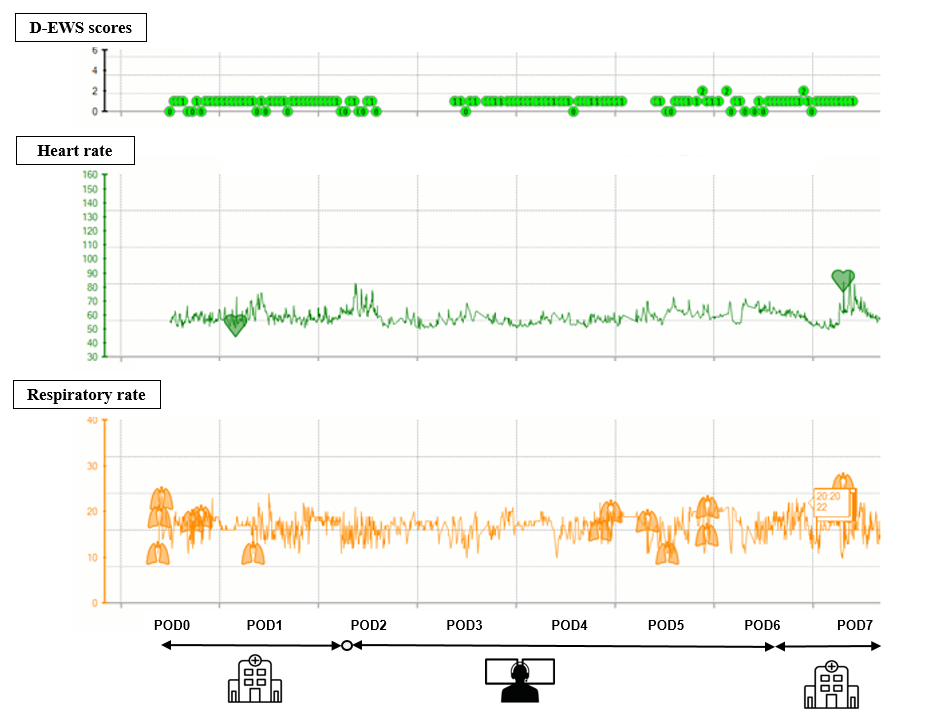


This is a Multimedia Appendix to a full manuscript published in the JMIR Perioperative Medicine. For full copyright and citation information see http://dx.doi.org/10.2196/jmir. 45113
