# Supplementary material for: Remote Home Monitoring of Continuous Vital Sign Measurements by Wearables in Patients Discharged After Colorectal Surgery: Observational Feasibility Study
Source: JMIR Perioper Med. 2023 May 5;6:e45113. doi: 10.2196/45113 (PMC10199380; doi:10.2196/45113)
Supplement: Multimedia Appendix 5 [file periop_v6i1e45113_app5.docx]

**Multimedia Appendix 5: Remarks of patients at the questionnaire (translated from Dutch)**

| *It does give confidence, also certainly the telephone contact with the nurse.* |
| --- |
| *I totally depend on the intervention.* |
| *Recovery at home is better. In the hospital there is always turmoil during the night.* |
| *I don't think I could have gone home earlier because I was waiting for blood tests. It was quite a heavy operation and I thought this was quick enough.* |
| *In the hospital should be the treatment. At home is the place for rest and recovery after surgery.* |
| *I do not feel any difference wearing the wearable sensor.* |
| *I do not feel any difference for wearing the home monitor.* |
| *I do sleep better at home.* |
| *East-West Home’s best.* |
| *At home is better, but in the hospital it is safer.* |
| *Recovery at home is better anyway.* |
| *Wearing the sensor: unnoticed. The telephone contact with the nurse was pleasant. However, I did not look forward to it or felt safer because of it.* |
| *I feel as safe as in hospital.* |
| *It is suitable if you feel good, not if you are uncertain about your recovery.* |
| *Being at home is more pleasant, not: in hospital professional help is quicker and nearby.* |
| *Everything is fine.* |
| *If there is an indication for continuous monitoring of vital signs, it seems to me that the patient should be monitored in the hospital and not at home.* |
| *It is understandable that the nurse during the night shift comes to the patient room to check or treat patients, but this causes the restless nights.* |
| *As far as I am concerned, the assessment of the vital sign trends should take place more often during the day.* |
| *I think it is a good intervention.* |
| *It is difficult to sleep sideways with the sensor.* |
| *After a few days the sensor starts itching. The sensor can cause bruised ribs.* |
| *After discharge some type of monitoring is nice.* |
| *It is difficult to sleep on your side with the sensor.* |
| *Make sure the sensor is in place when sleeping on the side.* |

This is a Multimedia Appendix to a full manuscript published in the JMIR Perioperative Medicine. For full copyright and citation information see http://dx.doi.org/10.2196/jmir. 45113
